# Paeoniflorin Modulates TREM-1/NF-κB/LXRα/ABCG1 Pathway to Improve Cholesterol Metabolism and Inflammation in Hyperlipidemic Rats

**DOI:** 10.3390/ijms27073039

**Published:** 2026-03-26

**Authors:** Ying Yang, Xiang Li, Dan-Li Tang, Bing Li, Si-Jia Wu, Hong-Xin Cao, Wen-Jing Zong, Hua-Min Zhang

**Affiliations:** 1 Institute of Basic Theory for Chinese Medicine, China Academy of Chinese Medical Sciences, Beijing 100700, China; yangyingwac@163.com (Y.Y.); lx15277912206@163.com (X.L.); 2 Experimental Research Centre of China Academy of Chinese Medical Sciences, Beijing 100700, China; tangdanli@merc.ac.cn; 3 Institute of Chinese Materia Medica, China Academy of Chinese Medical Sciences, Beijing 100700, China; libingtcm@163.com (B.L.); wusijia205@126.com (S.-J.W.); 4 China Association of Chinese Medicine, Beijing 100700, China; caohx898@163.com

**Keywords:** hyperlipidemic rat, paeoniflorin, multi-omics, inflammation, cholesterol metabolism

## Abstract

This study aimed to systematically elucidate the antihyperlipidemic mechanism of paeoniflorin, and we adopted an integrated multi-omics strategy to screen the key molecular targets and regulatory pathways involved in its action, followed by experimental validation to verify the potential regulatory effects of paeoniflorin on the screened targets and metabolic processes. Rats with high-fat diet-induced hyperlipidemia received paeoniflorin treatment. Liver histopathology was evaluated using hematoxylin–eosin and Oil Red O staining. Serum levels of total cholesterol, triglycerides, low-density lipoprotein cholesterol, high-density lipoprotein cholesterol, alanine aminotransferase, aspartate aminotransferase, alkaline phosphatase, total bile acids, activated partial thromboplastin time, prothrombin time, thrombin time, and fibrinogen were measured using a biochemical analyzer. Integrated multi-omics analyses were performed to investigate paeoniflorin’s lipid-lowering mechanism. Critical pathways and targets identified were validated using Western blotting. Paeoniflorin alleviated pathological liver damage in hyperlipidemic rats and improved blood lipid levels, coagulation function, and liver function markers. Multi-omics analyses verified that paeoniflorin downregulated the expression of TREM-1, TLR4, NF-κB, TNF-α, and IL-1β, thereby alleviating hepatic inflammation. Paeoniflorin also upregulated the expression of low-density lipoprotein receptors (LDLR), liver X receptor alpha (LXRα), and ATP-binding cassette subfamily G member 1 (ABCG1), while downregulating proprotein convertase subtilisin/kexin type 9 (PCSK9) expression, contributing to balanced cholesterol metabolism. Paeoniflorin normalized glycerophospholipid and branched-chain amino acid metabolism, which correlated with reduced inflammation and improved cholesterol metabolism. Paeoniflorin ameliorates hyperlipidemia through multitarget mechanisms, potentially by suppressing the TREM-1-TLR4-NF-κB signaling pathway to reduce inflammation and by regulating cholesterol metabolism via the PCSK9-LDLR and LXRα-ABCG1 pathways.

## 1. Introduction

Hyperlipidemia is a chronic disorder of lipid metabolism characterized by abnormally elevated serum lipid levels, including total cholesterol (TC), low-density lipoprotein cholesterol (LDL-C), and triglycerides (TG) [1]. In the United States, it is a major independent risk factor for cardiovascular and cerebrovascular diseases such as atherosclerosis, coronary heart disease, and ischemic stroke [2]. More than 100 million Americans have elevated LDL-C levels, putting them at a significantly increased risk of developing atherosclerotic cardiovascular disease [3]. Although statins, the current first-line treatment, demonstrate good efficacy, their long-term use is associated with adverse effects, such as liver and kidney dysfunction [4]. Therefore, it is imperative to explore novel, safer, and more effective therapeutic strategies. Traditional Chinese Medicine (TCM) has been increasingly evaluated for its efficacy in the treatment of hyperlipidemia. TCM can alleviate dyslipidemia and reduce adverse effects through multiple pathways [5,6,7], such as reducing endogenous cholesterol synthesis, modulating cholesterol transport, promoting hepatic cholesterol excretion, and regulating lipid-related transcription factors.

Paeoniflorin is derived from *Paeonia suffruticosa* Andr., *Paeonia lactiflora* Pall., and *Paeonia veitchii* Lynch and has been used in traditional medicine for over 2000 years. Modern pharmacological research demonstrates that paeoniflorin exhibits multitarget and multi-pathway biological activities, showing significant potential for the prevention and treatment of liver diseases [8]. Reported pharmacological effects include liver protection, alleviation of cholestasis, attenuation of liver fibrosis, prevention of non-alcoholic fatty liver disease, and inhibition of hepatocellular carcinoma, which involve multiple pathways [9,10,11,12,13]. Although paeoniflorin has been shown to exert significant antihyperlipidemic effects by modulating gut microbiota composition [14], its mechanism of action remains unclear. To date, the therapeutic efficacy of paeoniflorin in ameliorating dyslipidemia has not been systematically assessed across multiple key aspects, including its dose-dependent effects, specific molecular regulatory targets, and the crosstalk between its anti-inflammatory and lipid-modulating actions. Consequently, elucidating the molecular networks and mechanisms by which paeoniflorin modulates lipid metabolism will not only contribute to deciphering its pharmacodynamic basis, characterized by “multitarget, holistic regulation”, but also provide crucial scientific rationale for developing safe and effective lipid-lowering strategies derived from natural products.

Systematic biology and data-driven omics analysis methods, such as transcriptomics, proteomics, and metabolomics, play an indispensable role in decoding the interaction between TCM and diseases, offering comprehensive insight into their therapeutic mechanisms [15]. Multiomics technology is a comprehensive analytical approach that integrates high-throughput data derived from genomics, transcriptomics, proteomics, and metabolomics [16,17]. By consolidating multidimensional biomolecular datasets, researchers can systematically decipher complex regulatory networks within organisms under both physiological and pathological conditions. TCM holds unique advantages in treating complex diseases due to its multicomponent, multitarget, and multipathway synergistic actions [18]. However, the inherent complexity of its mechanism of action presents significant challenges for the precise elucidation of therapeutic targets and pathways. In this context, leveraging integrated multi-omics strategies to delve into multi-layered molecular alterations in the host following TCM intervention, identifying key regulatory targets and biomarkers, and reconstructing interacting signaling pathway networks have emerged as a new paradigm and powerful driving force, propelling the systematic elucidation of the multitarget synergistic mechanisms underlying therapeutic effects of TCM on diseases.

Dyslipidemia is a complex metabolic disorder characterized by abnormal serum lipid accumulation, and its pathogenesis is closely intertwined with chronic low-grade inflammation and impaired cholesterol metabolic homeostasis, with the two processes forming a mutually reinforcing vicious cycle [19,20]. Chronic inflammation is a key driver of dyslipidemia progression: persistent inflammatory stimulation disrupts the normal regulation of hepatic lipid metabolism, exacerbates lipid deposition in the liver and peripheral blood, and further impairs lipid clearance capacity [21]. In turn, disordered cholesterol metabolism can trigger and amplify the inflammatory response: abnormal accumulation of cholesterol and its metabolites can activate innate immune signaling pathways in immune and parenchymal cells, leading to excessive release of proinflammatory factors and the formation of a chronic inflammatory microenvironment [22,23]. The liver, as the core organ of cholesterol synthesis, uptake and excretion, is the key intersection where cholesterol metabolic disorder and inflammatory response interact and regulate each other in dyslipidemia [20]. Targeting the crosstalk between cholesterol metabolism and inflammation has thus become a critical biological strategy for the prevention and treatment of dyslipidemia and its associated cardiovascular complications [24]. In this study, we established a high-fat diet-induced hyperlipidemic rat model, and used a comprehensive multi-omics approach to comprehensively analyze the molecular and metabolic changes in hyperlipidemic rats after paeoniflorin intervention; And then we conducted targeted experimental validation to clarify the key biological processes mediated by paeoniflorin in ameliorating hyperlipidemia, so as to fully reveal its antihyperlipidemic biological mechanism.

## 2. Results

### 2.1. Paeoniflorin Reduced Liver Fat Accumulation in HFD-Induced Hyperlipidemia Rats

**Figure 1:**
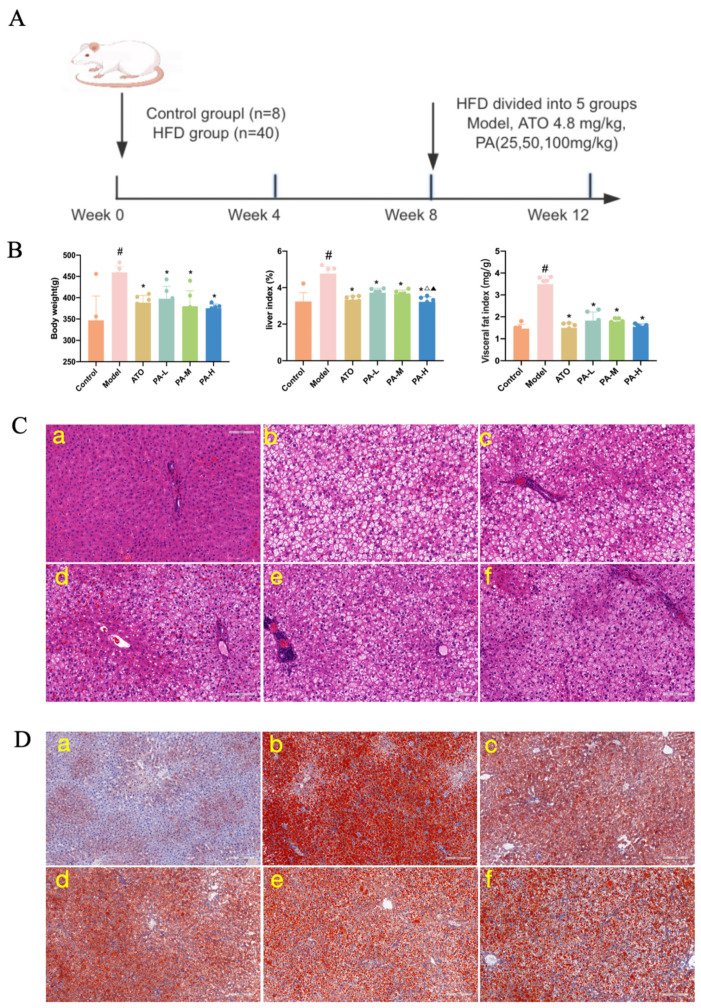
(**A**) Establishment of the rat model and groups. (**B**) Body weight, liver index, and visceral fat index. (**C**) HE staining of the liver (200×). (**D**) Oil Red O staining of the liver (100×). In comparison to the control group, ^#^ *p* < 0.05. In comparison to the model group, * *p* < 0.05. In comparison to the PA-L group, ^△^ *p* < 0.05. In comparison to the PA-M group, ^▲^ *p* < 0.05. (**a**): control group, (**b**): model group, (**c**): ATO group, (**d**): PA-L group, (**e**): PA-M group, (**f**): PA-H group.

As shown in Figure 1A, the model group exhibited markedly higher body weight and visceral fat index but a lower liver index, compared with the control group. Paeoniflorin administration reduced body weight and visceral fat index while increasing liver index. Furthermore, the high-dose paeoniflorin group exhibited a significantly greater increase in the liver index than both the low-dose and medium-dose paeoniflorin groups. As shown in Figure 1B,C, histological examination of the control group revealed distinct liver lobules with a well-organized architecture and plump and rounded hepatocytes. Conversely, the model group exhibited mild liver steatosis, characterized by variably sized circular vacuoles in the cytoplasm. Additionally, Oil Red O staining confirmed the presence of abundant red-stained lipid droplets in the hepatocytes of the model group. Compared to the model group, all treatment groups showed a reduction in cytoplasmic vacuolation, a significant decrease in ballooning degeneration of hepatocytes, and notably fewer red-stained lipid droplets in the cytoplasm.

### 2.2. Paeoniflorin Improved Biochemical Parameters in HFD-Induced Hyperlipidemia Rats

**Figure 2:**
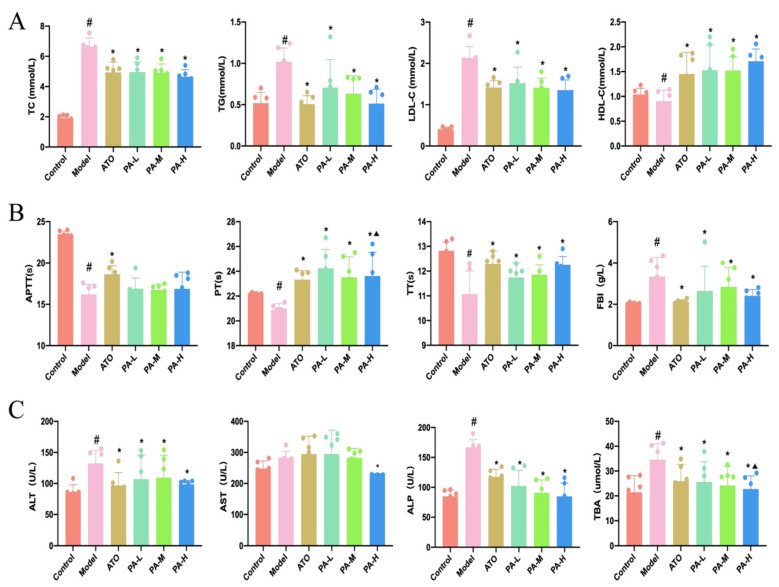
(**A**) Blood lipid (TC, TG, LDL-C, and HDL-C). (**B**) Coagulation function (APTT, PT, TT, and FIB). (**C**) Liver function (ALT, AST, ALP, and TBA). In comparison to the control group, ^#^ *p* < 0.05. In comparison to the model group, * *p* < 0.05. In comparison to the PA-M group, ^▲^ *p* < 0.05.

As depicted in Figure 2A, compared with the control group, rats in the model group exhibited significantly elevated serum TC, TG, and LDL-C levels, accompanied by a significant reduction in HDL-C. Treatment with paeoniflorin or atorvastatin calcium significantly reduced serum TG, TC, and LDL-C levels. Notably, paeoniflorin and ATO significantly increased HDL-C levels compared with the model group. The coagulation method was used to measure four coagulation parameters (Figure 2B). Relative to the control group, the model group exhibited shorter APTT, PT, and TT, along with elevated FIB levels. Conversely, the PA-L, PA-M, PA-H, and ATO groups showed increased PT and TT levels and reduced FIB levels, with ATO additionally increasing APTT levels. Notably, PT levels in the PA-H group were significantly higher than those in the PA-M group. Subsequently, we explored the effects of paeoniflorin on liver damage in HFD-fed rats. As outlined in Figure 2C, compared with the control group, the model group displayed significantly elevated serum ALT, ALP, and TBA levels, with AST levels showing an upward trend. Both ATO and paeoniflorin treatments significantly reduced ALT, ALP, and TBA levels. Furthermore, PA-H had a more significant TBA-lowering effect than PA-M and significantly reduced AST levels.

### 2.3. Transcriptomics Identified the Genes and Pathways Related to the Effect of Paeoniflorin Against Hyperlipidemia

**Figure 3:**
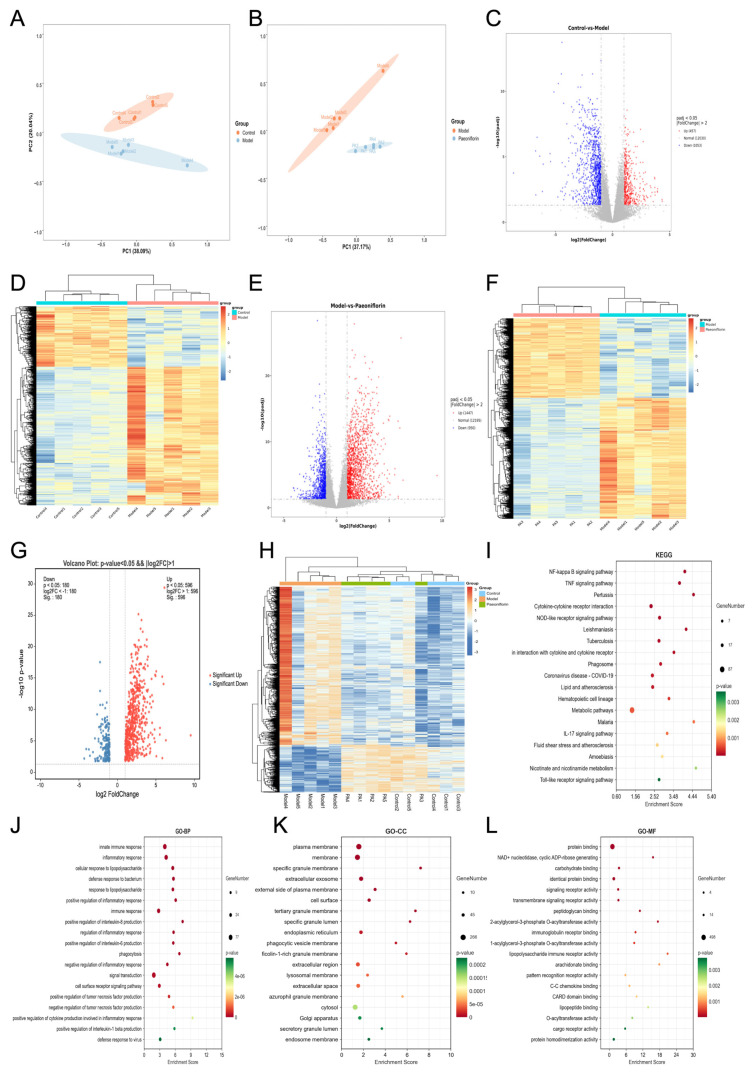
(**A**) PCA plot between control and model groups. (**B**) PCA plot between model and paeoniflorin groups. (**C**) Volcano plot of DEGs between control and model groups. (**D**) Clustered heatmap of DEGs between control and model groups. (**E**) Volcano plot of DEGs between model and paeoniflorin groups. (**F**) Clustered heatmap of DEGs between model and paeoniflorin groups. (**G**) Volcano plot of DEGs reversed by paeoniflorin intervention. (**H**) Clustered heatmap of DEGs reversed by paeoniflorin intervention. (**I**) KEGG enrichment analysis of DEGs reversed by paeoniflorin intervention. (**J**) GO-BP enrichment analysis of DEGs reversed by paeoniflorin intervention. (**K**) GO-CC enrichment analysis of DEGs reversed by paeoniflorin intervention. (**L**) GO-MF enrichment analysis of DEGs reversed by paeoniflorin intervention.

Principal component analysis (PCA) revealed distinct clustering and clear separation between the control and model groups, as well as between the model and paeoniflorin-treated groups (Figure 3A,B). Applying the threshold criteria of |log2^FoldChange^| > 1 and *p* < 0.05, 1967 DEGs were identified between the model and control groups, comprising 457 upregulated and 1053 downregulated genes (Figure 3C,D). A comparison between the paeoniflorin-treated and model groups yielded 2397 DEGs, of which 1447 were upregulated and 950 were downregulated (Figure 3E,F). Among the 1967 DEGs altered in the model group, 776 exhibited a significant reversal toward normal expression levels following paeoniflorin treatment (Figure 3G,H). Functional enrichment analysis indicated that these paeoniflorin-reverted genes were predominantly enriched in inflammatory signaling pathways, including NF-κB and toll-like receptor signaling pathways (Figure 3I–L).

### 2.4. Proteomics Identified the Protein and Pathways Related to the Effect of Paeoniflorin Against Hyperlipidemia

**Figure 4:**
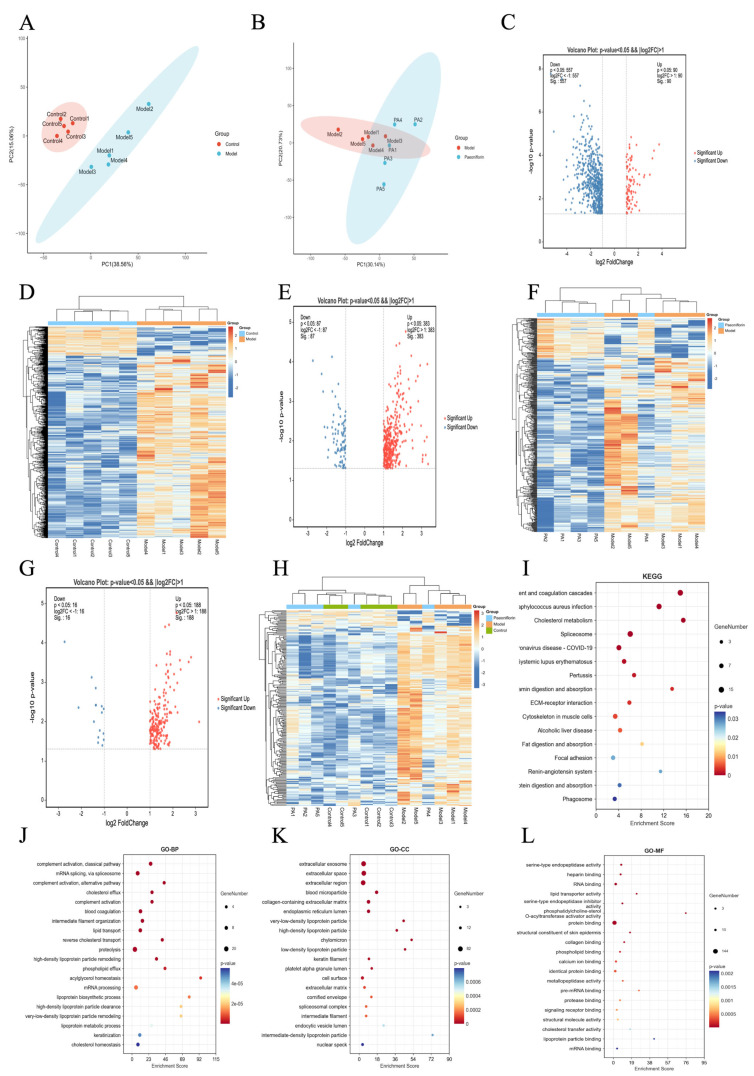
(**A**) PCA plot between control and model groups. (**B**) PCA plot between model and paeoniflorin groups. (**C**) Volcano plot of DEPs between control and model groups. (**D**) Clustered heatmap of DEPs between control and model groups. (**E**) Volcano plot of DEPs between the model and paeoniflorin groups. (**F**) Clustered heatmap of DEPs between the model and paeoniflorin groups. (**G**) Volcano plot of DEPs reversed by paeoniflorin intervention. (**H**) Clustered heatmap of DEPs reversed by paeoniflorin intervention. (**I**) KEGG enrichment analysis of DEPs reversed by paeoniflorin intervention. (**J**) GO-BP enrichment analysis of DEPs reversed by paeoniflorin intervention. (**K**) GO-CC enrichment analysis of DEPs reversed by paeoniflorin intervention. (**L**) GO-MF enrichment analysis of DEPs reversed by paeoniflorin intervention.

PCA revealed distinct intergroup separation and tight clustering across all experimental groups (Figure 4A,B). Applying the threshold criteria of |log2^FoldChange^| > 1 and *p* < 0.05, 647 DEPs were identified between the model and control groups, comprising 90 upregulated and 557 downregulated proteins (Figure 4C,D). A comparison of the paeoniflorin-treated and model groups yielded 470 DEPs, including 383 upregulated and 87 downregulated proteins (Figure 4E,F). Among the 647 DEPs dysregulated in the model group, 204 exhibited significant reversal toward control levels following paeoniflorin treatment (Figure 4G,H). KEGG and GO enrichment analyses indicated that these paeoniflorin-reverted proteins were predominantly enriched in metabolic pathways, particularly “cholesterol metabolism” and “reverse cholesterol transport” (Figure 4I–L).

### 2.5. Analysis of Gene-Protein Functional Module

**Figure 5:**
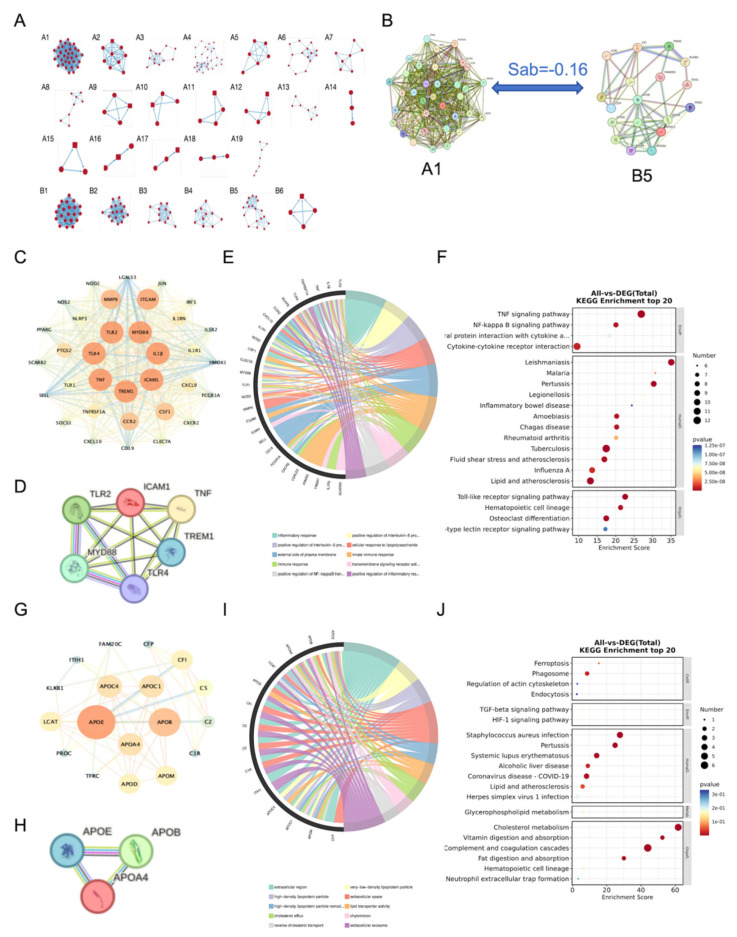
(**A**) Gene/protein modules reversed by paeoniflorin intervention. (**B**) SAB analysis between modules A1 and B5. (**C**) PPI network of module A1. (**D**) Core genes of module A1. (**E**) KEGG chord chart of module A1. (**F**) KEGG bubble chart of module A1. (**G**) PPI network of module B5. (**H**) Core genes of module B5. (**I**) KEGG chord chart of module B5. (**J**) KEGG bubble chart module B5.

Differentially expressed genes and proteins modulated by paeoniflorin were imported into the STRING database, and functional modules were identified using the MCODE plugin in Cytoscape 3.7.1. The reverted genes clustered into 19 distinct modules (A1–19), whereas the reverted proteins formed six modules (B1–6) (Figure 5A). Module interaction analysis using the separation score (SAB) revealed that SAB < 0 indicated a strong inter-module synergy. Notably, modules A1 and B5 exhibited the smallest SAB values (Figure 5B), demonstrating the highest cooperative interactions and the closest functional associations. Module A1 consisted of 34 nodes and 434 edges (Figure 5C), within which seven hub genes were identified: TNFα, IL1β, ICAM1, TLR4, TLR2, MYD88, and TREM1 (Figure 5D). Functional enrichment analysis revealed that these hub genes were primarily enriched in the TLR and NF-κB signaling pathways (Figure 5E,F). Module B5 comprised 18 nodes and 55 edges (Figure 5G), and the core proteins identified in this module were APOA4, APOB, and APOE (Figure 5H). Enrichment analysis indicated their predominant involvement in pathways related to cholesterol metabolism (Figure 5I,J).

### 2.6. Metabolomics Identified the Related Metabolites and Pathways of Paeoniflorin’s Anti-Hyperlipidemia Effect

**Figure 6:**
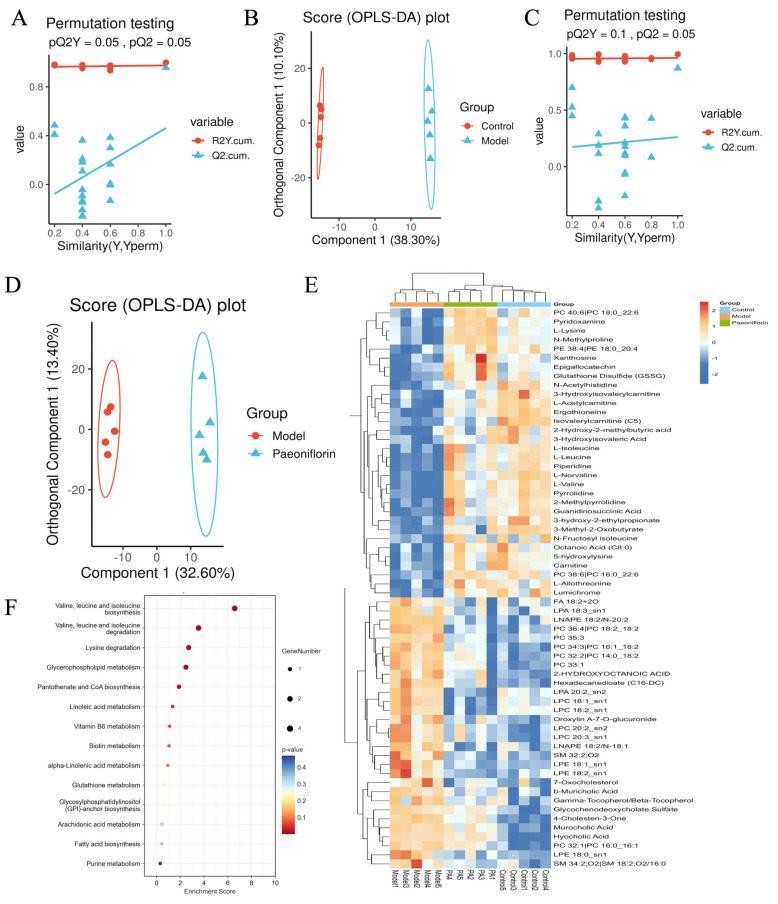
(**A**) Validation of the OPLS-DA between control and model groups. (**B**) OPLS-DA plot between control and model groups. (**C**) Validation of the OPLS-DA between control and model groups. (**D**) OPLS-DA plot between model and paeoniflorin groups. (**E**) Clustered heatmap of DEMs reversed by paeoniflorin intervention. (**F**) KEGG enrichment analysis of DEMs reversed by paeoniflorin intervention.

OPLS-DA analysis revealed a clear separation between the control and model groups, as well as between the paeoniflorin and model groups, indicating distinct metabolic profiles (Figure 6A–D). By applying the screening criteria of VIP > 1 and *p* < 0.05, 295 differential metabolites were identified between the model and control groups, comprising 142 upregulated and 153 downregulated metabolites in the model group. Among these 295 differential metabolites, 61 metabolites were significantly reversed to normal levels following paeoniflorin treatment (Figure 6E). These metabolites primarily consisted of glycerophospholipids (32.8%), followed by leucine, isoleucine, valine (13.1%), and fatty acids (8.2%) (Table 1). Specifically, glycerophospholipids in the model group, including phosphatidylcholines (PCs) such as PC 32:1|PC 16:0_16:1, PC 32:2|PC 14:0_18:2, PC 33:1, PC 34:3|PC 16:1_18:2, PC 35:3, and PC 36:4|PC 18:2_18:2, were significantly normalized by paeoniflorin treatment. Similarly, downregulated metabolites in the leucine, isoleucine, and valine metabolic pathways, including 3-hydroxy-2-ethylpropionate, 3-hydroxyisovaleric acid, 3-hydroxyisovalerylcarnitine, and L-isoleucine, were significantly restored. Pathway enrichment analysis further revealed that the metabolites reversed by paeoniflorin were predominantly enriched in valine, leucine, and isoleucine biosynthesis and degradation, as well as in glycerophospholipid metabolism (Figure 6F).

### 2.7. Effect of Paeoniflorin on Inflammation and Cholesterol Metabolism in Hyperlipidemic Rats

**Figure 7:**
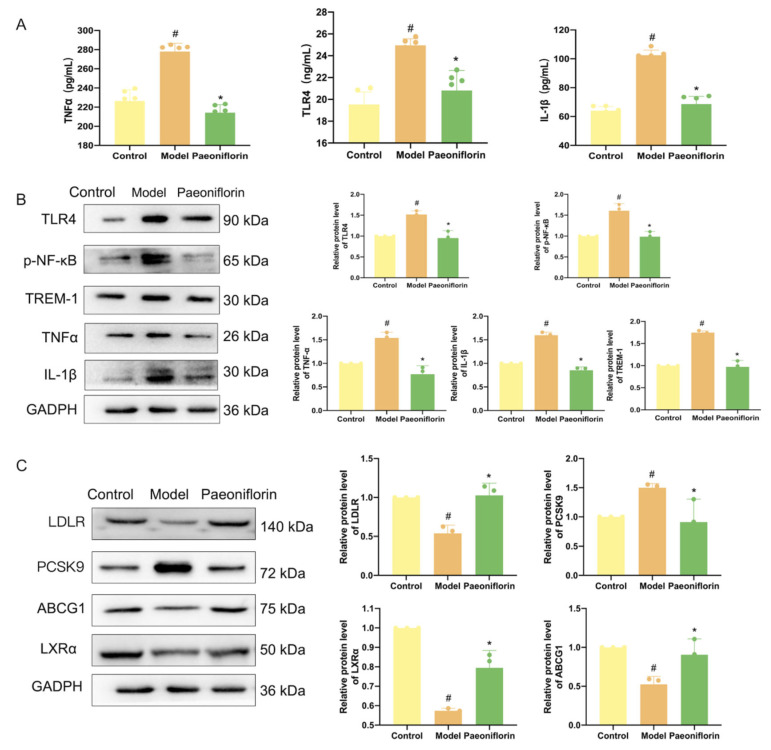
(**A**) The levels of inflammatory factors. (**B**) Protein expression of TREM-1, TLR4, p-p-NF-κB, TNFα, and IL-1β in liver., and (**C**) Protein expression of LDLR, PCSK9, LXRα and ABCG1 in liver. In comparison to the control group, ^#^ *p* < 0.05. In comparison to the model group, * *p* < 0.05.

As presented in Figure 7A,B, liver expression of TREM-1, TLR4, and p-NF-κB was increased in the model group compared with the control group. In contrast, paeoniflorin treatment markedly reduced the expression of these inflammatory mediators relative to the model group. IL-1β and TNFα are inflammatory factors downstream of p-NF-κB, which were also increased in the model group compared with the control group, but were decreased in the paeoniflorin group. Furthermore, paeoniflorin reduced the plasma levels of TLR4, TNF-α, and IL-1β.

As shown in Figure 7C, the LDLR-PCSK9 signaling axis regulates cholesterol uptake. In the model group, the expression of LDLR was significantly downregulated, whereas PCSK9 expression was upregulated, compared with the control group. Paeoniflorin treatment increased LDLR levels and reduced PCSK9 expression, thereby inhibiting cholesterol uptake. The LXRα-ABCG1 signaling axis, which promotes cholesterol efflux, was also impaired in the model group, as indicated by significantly reduced expression of LXRα, and ABCG1. In contrast, the expression of LXRα and ABCG1 in the paeoniflorin group was significantly upregulated, promoting cholesterol efflux. Overall, paeoniflorin boosts LDL-C clearance via the PCSK9-LDLR axis and activates cholesterol efflux and reverse cholesterol transport (RCT) through the LXRα-ABCG1 axis.

## 3. Discussion

Hyperlipidemia, also known as dyslipidemia, is characterized by elevated serum cholesterol and triglyceride levels. It is a major contributor to the high mortality rates associated with cardiovascular diseases and poses a serious threat to global health [25]. Therefore, the development of effective and safe therapeutic agents is urgently required. Previous studies have demonstrated that paeoniflorin effectively alleviates hyperlipidemia in atherosclerotic mice [26]. Furthermore, paeoniflorin treatment significantly reduces serum and liver cholesterol levels, liver steatosis, and cholesterol metabolites [27]. Despite these findings, the specific effects and underlying mechanisms of paeoniflorin on hyperlipidemia remain unclear. To address this gap, this study comprehensively investigated the ameliorative effects and mechanisms of action of paeoniflorin on hyperlipidemia in rats using integrated multi-omics sequencing.

Elevated serum levels of TC and TG are critical biochemical markers for assessing hyperlipidemia risk [28], as they both promote vascular endothelial injury and lipid deposition. Studies have indicated that LDL-C undergoes oxidative modification to form oxidized LDL, which induces apoptosis of vascular endothelial cells and activates macrophages, driving their transformation into foam cells. This process simultaneously triggers inflammatory signaling pathways, such as NF-κB, and promotes the release of proinflammatory cytokines, including IL-6 and TNF-α, thereby establishing a chronic inflammatory microenvironment [22]. Conversely, HDL-C reduces cholesterol deposition in the vascular wall by facilitating RCT, a mechanism that shuttles cholesterol from peripheral tissues to the liver for metabolism and excretion [29]. Our results demonstrated that paeoniflorin significantly improved the overall serum lipid profile by reducing TC, TG, and LDL-C in hyperlipidemic rats. Importantly, HDL-C levels were decreased in the model group but were significantly elevated by paeoniflorin treatment, indicating a favorable normalization of lipid metabolism rather than a detrimental effect. Since HDL-C was not elevated in the model, the observed increase supports a beneficial regulatory effect rather than impairment of reverse cholesterol transport. However, ApoA-I levels and HDL cholesterol efflux capacity were not evaluated in the present study, which represents a limitation to be addressed in future investigations. Because dyslipidemia can impair coagulation through multiple pathways [30], paeoniflorin also significantly prolonged APTT, PT, and TT, and reduced FIB levels, effectively reversing coagulation abnormalities in hyperlipidemic rats, the beneficial effects of paeoniflorin on coagulation may therefore occur indirectly through its lipid-lowering and anti-inflammatory actions. However, since platelet and endothelial markers were not measured in the present study, the precise mechanism remains to be further elucidated. Lipid droplet accumulation in the liver tissue leads to structural and functional alterations that can exacerbate hyperlipidemia [31]. Therefore, reducing fat accumulation in the liver may prevent hyperlipidemia. In this study, HFD induced lipid droplet accumulation in rat liver tissue and abnormal transaminase activity, as evidenced by significantly elevated levels of ALT, AST, ALP, and TBA. Paeoniflorin markedly ameliorated HFD-induced liver vacuolar degeneration and adipose tissue pathology, reduced lipid droplet formation, and lowered serum ALT, AST, ALP, and TBA levels, indicating significant hepatoprotective effects against HFD-induced liver injury.

Long-term HFD or energy surplus can cause excessive expansion and dysfunction of adipose tissue, triggering local hypoxia and cellular stress, which in turn induces a systemic, chronic, low-grade inflammatory state [19]. Relevant studies have confirmed that elevated blood lipid levels in patients with hyperlipidemia are often accompanied by increased serum inflammatory cytokines [32]. Transcriptomic analysis indicates that the lipid-lowering effect of paeoniflorin may be associated with its regulation of TLR and NF-κB signaling pathways. TREM-1, a member of the TREM family within the immunoglobulin (Ig) superfamily of activating receptors, participates in the innate inflammatory response. Previous studies demonstrated that TREM-1 is a key regulator of Kupffer cell activation and exacerbates both acute and chronic inflammatory responses in liver diseases [23,33]. Additionally, TREM-1 can amplify TLR4-mediated proinflammatory signaling [34]. Aberrant overactivation of the TLR4/NF-κB signaling pathway in hyperlipidemia has been confirmed to correlate with disease severity [21]. This study measured the mRNA and protein levels of TREM-1, TLR4, NF-κB, TNF-α, and IL-1β in liver tissue. The results indicated that paeoniflorin significantly decreased the mRNA and protein expression of TREM-1, TLR4, NF-κB, TNF-α, and IL-1β in the livers of hyperlipidemic rats. Furthermore, circulating levels of TNF-α and IL-1β were also reduced, indicating that paeoniflorin exerts anti-inflammatory effects by inhibiting the activation of the TREM-1-TLR4-NF-κB pathway, thereby reducing the expression of the inflammatory cytokines TNF-α and IL-1β.

The liver, as the central regulatory organ for cholesterol metabolism [20], plays a pivotal role in balancing cholesterol biosynthesis and clearance. Disruption of this balance is a critical pathological basis for hyperlipidemia development [35]. Proteomic analysis indicated that the lipid-lowering effect of paeoniflorin may be closely associated with its regulation of cholesterol metabolism. Multiple proteins, rate-limiting enzymes, and transcriptional regulators act synergistically to maintain cholesterol metabolism in the liver. Among these, cholesterol uptake plays a pivotal role in cell membrane synthesis and storage or conversion [36], whereas the efficiency of cholesterol efflux determines whether cholesterol accumulates within the body, thereby impacting overall physiological function and health [37]. This study evaluated the mechanism by which paeoniflorin regulates cholesterol metabolism, with a specific focus on cholesterol uptake, efflux, and utilization. LDLR, PCSK9, LXRα, and ABCG1 are key regulators of these processes in the liver. LDLR is the primary receptor responsible for reducing plasma LDL-C levels by mediating cholesterol uptake from very-LDL, LDL, and chylomicrons, thereby maintaining plasma cholesterol homeostasis [20]. PCSK9 degrades LDLR, thereby reducing its expression. Upon binding to the LDLR, PCSK9 induces lysosomal degradation, which in turn inhibits LDL uptake [38]. The LXRα-ABCG1 signaling pathway is a key regulator of liver cholesterol [39], facilitating the conversion of cholesterol into bile acids and effectively reducing liver cholesterol content. Activation of this pathway increases cholesterol conversion and efflux and prevents accumulation, contributing to the treatment of hyperlipidemia [40]. In this study, paeoniflorin significantly upregulated the expression of LDLR, LXRα, and ABCG1 protein in the liver of hyperlipidemic rats and markedly downregulated PCSK9 expression. This indicates that paeoniflorin enhances cholesterol uptake by regulating the PCSK9-LDLR pathway and promotes cholesterol utilization and efflux by activating the LXRα-ABCG1 signaling pathway.

Studies have demonstrated a close association between hyperlipidemia and disturbances in glycerophospholipid metabolism [24]. Glycerophospholipid metabolites, specifically PC and LPC, can activate the TLR4 signaling pathway, promote the release of inflammatory cytokines, and amplify the inflammatory cascade [41,42]. Conversely, TLR4-mediated proinflammatory signaling can also increase choline uptake and de novo PC synthesis in macrophages. In this study, paeoniflorin treatment reduced the levels of the PC and LPC, accompanied by a decrease in the levels of inflammatory factors in the liver and circulation, thereby mitigating the inflammatory response in hyperlipidemic rats. Leucine, isoleucine, and valine metabolism, which collectively constitute branched-chain amino acid (BCAA) metabolism, are closely linked to cholesterol metabolism [43]. BCAAs have been revealed to ameliorate liver lipid accumulation and dyslipidemia in HFD fed mice. Consistent with previous studies, the results indicate abnormal BCAA metabolism in hyperlipidemic rats [44]. Paeoniflorin intervention elevated the levels of L-isoleucine, L-leucine, and L-valine, key regulators that may alleviate hyperlipidemia by regulating cholesterol metabolism. Nonetheless, further experimental studies are needed to confirm this mechanism.

**Figure 8:**
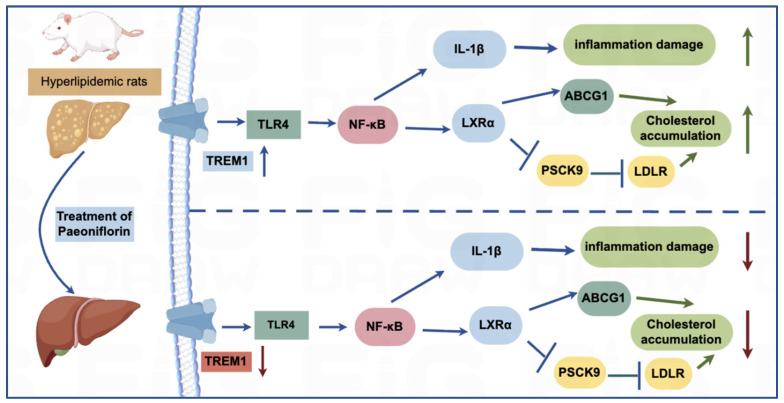
Paeoniflorin modulates TREM-1/NF-κB/LXRα/ABCG1 pathway to improve cholesterol metabolism and inflammation in Hyperlipidemic rat.

Early studies have confirmed that paeoniflorin reduces serum lipid levels, alleviates hepatic steatosis and exerts anti-inflammatory effects in hyperlipidemic models, primarily by regulating cholesterol metabolism and modulating gut microbiota composition [14,27], but these investigations were largely limited to phenotypic observations or single-dimensional molecular analyses, with the systemic regulatory network underlying its effects remaining poorly defined. The present study advances this field by adopting an integrated multi-omics strategy to systematically profile the molecular and metabolic changes induced by paeoniflorin in hyperlipidemic rats. The results of this study show that paeoniflorin exerts its effects through multiple pathways: the inhibition of TREM-1-TLR4-NF-κB inflammatory axis can reduce the inflammatory-mediated downregulation of LXRα/ABCG1 and LDLR, thereby promoting cholesterol metabolism; the normalization of glycerophospholipid metabolism can inhibit the TLR4/NF-κB pathway activated by PC/LPC, further weakening the inflammatory response; the restoration of BCAA metabolism can improve liver lipid synthesis and oxidation, synergizing with the regulation of cholesterol transport-related molecules to reduce hepatic lipid accumulation. This multi-axis, multi-target regulatory network is the core of paeoniflorin’s “holistic regulation” antihyperlipidemic effect, which is consistent with the characteristics of traditional Chinese medicine active ingredients in treating complex diseases (Figure 8).

While the integrated strategy of multi-omics screening, Western blotting validation, and phenotypic assessment employed in this study reasonably supports a potential regulatory association between paeoniflorin and key pathways governing inflammation and cholesterol metabolism, the findings merely reflect a correlative relationship rather than definitive causal linkage. This is attributable to the lack of functional validation using pathway-specific inhibitors, gene manipulation techniques, or in vitro cellular models; subsequent investigations should incorporate such functional assays, including rescue experiments, to rigorously confirm the mechanistic causality. Despite these constraints, the findings hold considerable translational significance: they identify core regulatory targets of paeoniflorin, furnishing novel molecular insights for the development of antihyperlipidemic agents. Furthermore, the integrated multi-omics approach utilized herein offers a valuable paradigm for modern mechanistic investigations into the bioactive components of traditional Chinese medicine.

## 4. Materials and Methods

### 4.1. Establishment of the Rat Model and Groups

Animal use in this study was approved by the Institutional Ethics Committee of China Academy of Chinese Medical Sciences (approval number: IBTCMCACMS21-2406-05; approval date: 24 June 2024). Female Sprague-Dawley rats (*n* = 50; 180 ± 20 g) were provided by Si Pei Fu Biotechnology Co., Ltd. [SCXK (Beijing) 2024-0001, Beijing, China]. After 7 days of adaptive feeding, 48 rats were randomly divided into two groups: the control group (*n* = 8), which was fed an ordinary maintenance diet, and the remaining 40 rats, which were fed a high-fat diet. After 8 weeks of continuous feeding, blood lipid and coagulation function indices were measured. The model was considered successful if TC and TG levels were significantly higher than those of the rats in the control group. Successful model rats were then randomly divided into the five groups (*n* = 8 each): Model group, atorvastatin calcium group (ATO, 4.8 mg/kg), paeoniflorin (98.21% purity, Shaanxi Xintianyu Biotechnology Co., Ltd., Xi’an, China) groups at low (PA-L, 25 mg/kg), medium (PA-M, 50 mg/kg), and high (PA-H, 100 mg/kg) doses, this dose is a safe dose for rats [45]. The rats were continuously administered the drug for 4 weeks. Rats in the control and model groups received the same amount of purified water, as presented in Figure 1A.

### 4.2. Assessment of Liver Morphology

The liver was dehydrated, embedded in paraffin, and sectioned at a thickness of 4 µm using a microtome. The sections were deparaffinized, rinsed, stained with hematoxylin and eosin (HE) (Wuhan Servicebio Technology Co., Ltd., Wuhan, China), washed, dehydrated through a graded ethanol series, cleared in xylene, and mounted with neutral balsam to observe histopathological changes under a light microscope. For lipid visualization, liver tissues were embedded in OCT compound, sectioned at a thickness of 8 µm using a cryostat, and stained with Oil Red O (Wuhan Servicebio Technology Co., Ltd., Wuhan, China). Lipid accumulation was then assessed under a light microscope.

### 4.3. Detection of Coagulation Function, Serum Lipid, Liver Function and Inflammatory Factors

The prothrombin time (PT) activated partial thromboplastin time (APTT), thrombin time (TT), and fibrinogen (FIB) levels (Beijing Zhongchi Weiye Technology Development Co., Ltd., Beijing, China) were measured using a biochemical analyzer. TC, TG, LDL-C, high-density lipoprotein cholesterol (HDL-C), alanine aminotransferase (ALT), aspartate aminotransferase (AST), total bile acids (TBA), and alkaline phosphatase (ALP) (Beijing Beijian-Xinchuangyuan Biotechnology Co., Ltd., Beijing, China) were also measured using a biochemical analyzer; The levels of Tumor Necrosis Factor-alpha (TNF-α), Interleukin-1 beta (IL-1β), Toll-Like Receptor 4 (TLR4), and Toll-Like Receptor 2 (TLR2) (both, Jiangsu Enzyme Standard Biotechnology Co., Taixing, China) were determined using enzyme-linked immunoassay kits.

### 4.4. Multi-Omics Sequencing

#### 4.4.1. Whole Blood Transcriptome Sequencing

Five PAXgene tubes were randomly selected from each group and thawed. Total RNA was extracted from whole blood using TRIzol reagent. For transcriptome analysis, mRNA was enriched with oligo(dT) beads, fragmented and subjected to first-strand cDNA synthesis and adapter ligation. The resulting fragments were size-selected, and the libraries were enriched through PCR amplification. Library concentrations were quantified using a QuantiFluor dsDNA system. Libraries were pooled in equimolar ratios based on the quantification data and loaded onto flow cells. Bridge amplification was performed using a cBot cluster generation system to generate clusters, and sequencing was carried out on an Illumina platform. Raw sequencing data underwent quality control using fastp v1.1.0 software. Gene and transcript expression levels were quantified as read counts using featureCounts. Differentially expressed genes (DEGs) were identified from the read count matrix using differential expression analysis software, with a significance threshold of |log_2_^fold change^| > 1 and *p* < 0.05. Kyoto Encyclopedia of Genes and Genomes (KEGG) and Gene Ontology (GO) databases were used for pathway enrichment analysis of the DEGs.

#### 4.4.2. Plasma Astral Proteomic Sequencing

Five rat plasma samples were randomly selected from each group and thawed. The samples were centrifuged at 4 °C, 15,000× *g* for 10 min, after which the precipitate was removed and the supernatant transferred to a new centrifuge tube. Highly abundant proteins were removed as described in the Pierce™ Top 14 Abundant Protein Depletion Spin Columns Kit (Thermo Fisher Scientific Inc., Waltham, MA, USA). Protein concentrations were determined using the bicinchoninic acid (BCA) method. Chromatographic separation was performed using the nanoflow rate Vanquish Neo system (Thermo Fisher Scientific Inc., Waltham, MA, USA), after which the samples were subjected to data-independent acquisition (DIA) mass spectrometry using an Astral high-resolution mass spectrometer (Thermo Scientific). The analysis was conducted in positive ion mode with a precursor ion scan range of 380–980 *m*/*z* and a primary mass spectrometry resolution of 240,000 at 200 *m*/*z*. The Normalized automatic gain control (AGC) Target was set at 500% with a maximum injection time(IT) of 5 ms. MS2 employed the DIA data acquisition mode with 299 scan windows, an isolation window of 2 *m*/*z*, high-energy collision dissociation (HCD) Collision Energy set at 25 eV, normalized AGC target at 500%, and a maximum IT of 3 ms. DIA data were processed using DIA-NN 2.3.2 software. Differentially expressed proteins (DEPs) were identified from the read count matrix using differential expression analysis software, with a significance threshold of |log_2_^fold change^| > 1 and *p* < 0.05. Functional enrichment analyses of DEPs were performed, including GO and KEGG.

#### 4.4.3. Constructing Gene-Protein Co-Expression Network

Differentially expressed genes and proteins reversed by paeoniflorin intervention were imported into the STRING database to construct a protein–protein interaction (PPI) network. Modules within the PPI network were identified using the MCODE plugin, a clustering algorithm-based tool in Cytoscape.

The S_AB_ score [46] was used to determine the relationship between the genes and protein modules. The network proximity of gene module A and protein module B was calculated using the shortest distance.

$$S_{AB}=\langle d_{AB}\rangle -\frac{<d_{AA}>+<d_{BB}>}{2}$$

Here, <d_AA_> and <d_BB_> represent the shortest distance within the interactome of each module, while <d_AB_> denotes the shortest distance between module A and B. An S_AB_ < 0 indicates that the targets of the two modules are in the same neighborhood, suggesting potential pathobiological and clinical similarities.

Using the S_AB_ score, the most tightly interconnected gene and protein modules were selected. PPI networks for these modules were then constructed, visualized, and analyzed in Cytoscape to screen core genes and proteins.

#### 4.4.4. Plasma Non-Targeted Metabolomics Sequencing

Plasma samples from five rats were randomly selected from each group and thawed. Briefly, 10 μL of each sample was mixed into the pool quality control (PQC) samples, vortexed, and mixed thoroughly. The extraction solvent (methanol pre-cooled for more than half an hour at −80 °C) was used as the blank solution, and 20 μL of serum, 20 μL of PQC, and 20 μL of blank solution were placed in a 2 mL centrifuge tube. Then 20 μL of internal standard working solution and 110 μL of extraction solvent (methanol pre-cooled for more than half an hour at −80 °C) were added. The solution was vortexed and oscillated for 1 min, incubated at −20 °C for more than 30 min, and centrifuged at 14,000× *g* at 4 °C for 10 min. The resulting supernatant was transferred to a new 2 mL centrifuge tube, vacuum freeze-dried, and redissolved in 100 μL of 10% methanol/90% aqueous solution (constant volume solution). The reconstituted sample was vortexed for 30 s, ultrasonicated for 1 min at 14,000× *g*, centrifuged at 4 °C for 10 min, and finally transferred to an injection vial for mass spectrometry analysis. Chromatographic separation was performed using an ACQUITY UPLC HSS T3 column (1.7 μm × 2.1 mm × 100 mm) with a mobile phase consisting of 0.1% formic acid aqueous solution (A) and 0.1% formic acid/acetonitrile/methanol solution gradient elution (0–3 min, 10–40% A; 3–5 min, 40–95% A; 5–8 min, 95–100% A; 8–10 min, 100% A; 10–10.6 min, 10% A; 10.6 min, 10% A; 10.6 min, 10% A), at a flow rate 0.25 mL·min^–1^, a column temperature 40 °C, and an injection volume 5 μL. Samples were analyzed on a Q Exactive HF-X (QE-HF-X) mass spectrometer (Thermo Fisher Scientific Inc., Waltham, MA, USA) with a heated electrospray ion source. The mobile phase consisted of 0.1% formic acid aqueous solution (A) and 0.1% acetonitrile/methanol solution with gradient elution (0–3 min, 10–40% B; 3–5 min, 40–95% B; 5–8 min, 95–100% B; 8–10 min, 100% B; 10–10.5 min, 10% B), at a flow rate 0.25 mL/min, followed by a 3 min equilibration. All data were tested in full scan using positive and negative ion switching modes, while PQC samples were also tested in full scan/ddMS2 mode to obtain the MS2 fragmentation information required for metabolite identification and annotation. The full scan was set as follows: resolution 60,000; AGC target was 1 × 10^6^; maximum IT 100 ms; and scanning range, 60–900 *m*/*z*. For full scan/ddMS2 (DDA), the first 20 MS/MS spectra (dd-MS2) at a resolution of 15,000 were generated using an AGC target of 2 × 10^5^, maximum IT of 25 ms, and (N)CE/stage NCE of 10, 40, and 80 V.

Metabolites were detected and identified by searching online and internal databases using the MS-dial software (version 5.1.230912). Based on the Human Metabolome Database (HMDB) and KEGG, all detected metabolites were annotated, functionally defined, classified, and visualized using the ggplot2 package (version 3.4.4). Log-transformed raw data were analyzed using multifactor statistical methods in the ropls R package 4.4.2, including orthogonal partial least square-discriminant analysis (OPLS-DA) and variable importance in projection (VIP) obtained by the OPLS-DA model. Differentially expressed metabolites (DEMs) between groups were screened using VIP > 1 and *p* < 0.05. Metabolic pathway enrichment was then performed using the MetaboAnalyst software (version 5.0).

### 4.5. Western Blotting

The expression of triggering receptor expressed on myeloid cells 1 (TREM-1), toll-like receptor 4 (TLR4), nuclear factor kappa-light-chain-enhancer of activated B cells (NF-κB), TNF-α (tumor necrosis factor-alpha), Interleukin-1 beta (IL-1β), low-density lipoprotein receptors (LDLR), proprotein convertase subtilisin/kexin type 9 (PCSK9), liver X receptor alpha (LXRα), and ATP-binding cassette subfamily G member 1 (ABCG1) in the liver was analyzed using Western blotting (Proteintech Group, Inc., Wuhan, China). Total liver and myocardial proteins were extracted using cell and tissue lysis buffers, a protease inhibitor cocktail, and a phosphatase inhibitor cocktail (APPLYGEN, Beijing, China). Proteins were quantified using a BCA protein assay kit (YEASEN, Shanghai, China). Proteins were separated using 8% sodium dodecyl sulfate-polyacrylamide gel electrophoresis and subsequently transferred onto PVDF membranes (Bio-Rad Laboratories, Inc., Hercules, DE, USA). Membranes were blocked in 5% skim milk at room temperature for 1 h, then incubated with primary antibodies overnight at 4 °C. The membranes were then incubated with secondary antibodies (Proteintech Group, Inc., Wuhan, China) for 1 h. Finally, the membranes were visualized using an enhanced chemiluminescence kit (Proteintech Group, Inc., Wuhan, China).

### 4.6. Statistical Analysis

The experimental results are expressed as mean ± standard deviation. Statistical analyses were performed, and charts were drawn using GraphPad Prism software (version 8). One-way analysis of variance (ANOVA) was used to compare groups. For uniform variance, the least significant difference test was used, whereas the Kruskal–Wallis test was used for non-uniform variance. Statistical significance was determined when the *p*-value was <0.05.

## 5. Conclusions

Integrated multi-omics analyses and experimental verification reveal that paeoniflorin ameliorates hyperlipidemia through multitarget mechanisms. These included suppressing inflammation via the TREM-1-TLR4-NF-κB axis, restoring cholesterol homeostasis through PCSK9-mediated uptake and LXRα-driven efflux, and regulating glycerophospholipid and BCAA metabolism. These findings provide evidence that paeoniflorin is a candidate drug for the treatment of hyperlipidemia and its associated complications.

## Figures and Tables

**Table 1:** Paeoniflorin can significantly regulate metabolites.

No.	Metabolite	Category	Trend of Change	
			Model/ Control	PA/ Model
1	PC 32:1|PC 16:0_16:1	Glycerophospholipid	↑	↓
2	PC 32:2|PC 14:0_18:2	Glycerophospholipid	↑	↓
3	PC 33:1	Glycerophospholipid	↑	↓
4	PC 34:3|PC 16:1_18:2	Glycerophospholipid	↑	↓
5	PC 35:3	Glycerophospholipid	↑	↓
6	PC 36:4|PC 18:2_18:2	Glycerophospholipid	↑	↓
7	PC 38:6|PC 16:0_22:6	Glycerophospholipid	↓	↑
8	PC 40:6|PC 18:0_22:6	Glycerophospholipid	↓	↑
9	LPC 18:1_sn1	Glycerophospholipid	↑	↓
10	LPC 18:2_sn1	Glycerophospholipid	↑	↓
11	LPC 20:2_sn2	Glycerophospholipid	↑	↓
12	LPC 20:3_sn1	Glycerophospholipid	↑	↓
13	LPE 18:0_sn1	Glycerophospholipid	↑	↓
14	LPE 18:1_sn1	Glycerophospholipid	↑	↓
15	LPE 18:2_sn1	Glycerophospholipid	↑	↓
16	PE 38:4|PE 18:0_20:4	Glycerophospholipid	↓	↑
17	LPA 18:3_sn1	Glycerophospholipid	↑	↓
18	LPA 20:2_sn2	Glycerophospholipid	↑	↓
19	LNAPE 18:2/N-18:1	Glycerophospholipid	↑	↓
20	LNAPE 18:2/N-20:2	Glycerophospholipid	↑	↓
21	3-hydroxy-2-ethylpropionate	Leucine, Isoleucine, and Valine Metabolism	↓	↑
22	3-Hydroxyisovaleric Acid	Leucine, Isoleucine, and Valine Metabolism	↓	↑
23	3-Hydroxyisovalerylcarnitine	Leucine, Isoleucine, and Valine Metabolism	↓	↑
24	3-Methyl-2-Oxobutyrate	Leucine, Isoleucine, and Valine Metabolism	↓	↑
25	Isovalerylcarnitine (C5)	Leucine, Isoleucine, and Valine Metabolism	↓	↑
26	L-Isoleucine	Leucine, Isoleucine, and Valine Metabolism	↓	↑
27	L-Leucine	Leucine, Isoleucine, and Valine Metabolism	↓	↑
28	L-Valine	Leucine, Isoleucine, and Valine Metabolism	↓	↑
29	2-Methylpyrrolidine	Natural Product/Food/Plant	↓	↑
30	Epigallocatechin	Natural Product/Food/Plant	↓	↑
31	Ergothioneine	Natural Product/Food/Plant	↓	↑
32	Lumichrome	Natural Product/Food/Plant	↓	↑
33	N-Fructosyl isoleucine	Natural Product/Food/Plant	↓	↑
34	Oroxylin A-7-O-glucuronide	Natural Product/Food/Plant	↑	↓
35	Piperidine	Natural Product/Food/Plant	↓	↑
36	Pyrrolidine	Natural Product/Food/Plant	↓	↑
37	β-Muricholic Acid	Bile Acid	↑	↓
38	Hyocholic Acid	Bile Acid	↑	↓
39	Murocholic Acid	Bile Acid	↑	↓
40	Glycochenodeoxycholate Sulfate	Bile Acid, sulfated	↑	↓
41	FA 18:2+2O	Fatty Acid Metabolism	↑	↓
42	Carnitine	Fatty Acid Metabolism	↓	↑
43	L-Acetylcarnitine	Fatty Acid Metabolism	↓	↑
44	SM 32:2;O2	Sphingolipid Metabolism	↑	↓
45	SM 34:2;O2|SM 18:2;O2/16:0	Sphingolipid Metabolism	↑	↓
46	4-Cholesten-3-One	Sterol	↑	↓
47	7-Oxocholesterol	Sterol	↑	↓
48	5-hydroxylysine	Lysine Metabolism	↓	↑
49	L-Lysine	Lysine Metabolism	↓	↑
50	Guanidinosuccinic Acid	Urea cycle; Arginine and Proline Metabolism	↓	↑
51	N-Methylproline	Urea cycle; Arginine and Proline Metabolism	↓	↑
52	L-Allothreonine	Other Amino Acid Metabolism	↓	↑
53	L-Norvaline	Other Amino Acid Metabolism	↓	↑
54	N-Acetylhistidine	Histidine Metabolism	↓	↑
55	Octanoic Acid (C8:0)	Medium Chain Fatty Acid	↓	↑
56	Gamma-Tocopherol/Beta-Tocopherol	Tocopherol Metabolism	↑	↓
57	Pyridoxamine	Vitamin B6 Metabolism	↓	↑
58	Hexadecanedioate (C16-DC)	Fatty Acid, Dicarboxylate	↑	↓
59	2-HYDROXYOCTANOIC ACID	Fatty Acid, Monohydroxy	↑	↓
60	Glutathione Disulfide (GSSG)	Glutathione Metabolism	↓	↑
61	Xanthosine	Purine Metabolism, (Hypo)Xanthine/Inosine containing	↓	↑

↑ upregulated; ↓ downregulated.

## Data Availability

The original contributions presented in this study are included in the article. Further inquiries can be directed to the corresponding authors.

## Glossary

ABCG1	ATP-binding cassette subfamily G member 1
ALP	alkaline phosphatase
ALT	alanine aminotransferase
AST	aspartate aminotransferase
DEGs	Differentially expressed genes
DEMs	Differentially expressed metabolites
DEPs	Differentially expressed proteins
FIB	fibrinogen
GO	gene ontology
HDL-C	high-density lipoprotein cholesterol
HE	hematoxylin and eosin
IL-1β	interleukin-1β
KEGG	Kyoto encyclopedia of genes and genomes
LDL-C	low-density lipoprotein cholesterol
LDLR	Low-density lipoprotein receptors
LXRα	liver X receptor alpha
NF-κB	nuclear factor kappa-light-chain-enhancer of activated B cells
OPLS-DA	orthogonal partial least square-discriminant analysis
PCSK9	proprotein convertase subtilisin/kexin type 9
PPI	protein–protein interaction
PT	prothrombin time
TBA	total bile acids
TC	total cholesterol
TCM	Traditional Chinese Medicine
TG	triglycerides
TLR2	toll-like receptor 2
TLR4	toll-like receptor 4
TNF-α	tumor necrosis factor-alpha
TREM-1	triggering receptor expressed on myeloid cells 1
TT	thromboplastin time
VIP	variable importance in projection
WB	Western blotting
